# The Rosacea-specific Quality-of-Life instrument (RosQol): Revision and validation among Chinese patients

**DOI:** 10.1371/journal.pone.0192487

**Published:** 2018-02-28

**Authors:** Yuxuan Deng, Qinqin Peng, Sai Yang, Dan Jian, Ben Wang, Yingxue Huang, Hongfu Xie, Ji Li

**Affiliations:** Department of Dermatology, Xiangya Hospital, Central South University, Changsha, Hunan Province, China; Jilin University, CHINA

## Abstract

Rosacea is a common chronic facial disorder that affects patients' health-related quality of life; the only questionnaire designed specifically for rosacea is the Rosacea-specific Quality-of-Life instrument (RosQol). However, the questionnaire has not been validated among Chinese patients. This study aimed to validate the Chinese version of the RosQol. First, we translated the questionnaire into Chinese. Then, rosacea patients completed the RosQol and Dermatology Life Quality Index, indicating the disease's impact on their lives. We also collected patients' demographic and clinical data, including symptom self-evaluation scores and rosacea severity scores. Internal consistency was determined by using Cronbach's alpha, test-retest reliability, and Spearman's correlation. Criterion-related validity and internal construct validity were also determined. Most RosQol items showed good internal consistency. However, items 13 and 19 were not sufficiently sensitive for use in the Chinese population; we deleted them and constructed the adjusted Chinese-version RosQol, which had good reliability and validity. When patients' clinical symptoms changed, the scores on the relevant dimensions of the adjusted RosQol also changed. Some RosQol items were not suitable for use in the Chinese sample. The adjusted Chinese-version RosQol was easy to complete, well received by patients, and demonstrated acceptable validity and reliability.

## Introduction

Rosacea is a common, chronic disorder of the facial skin blood vessels and pilosebaceous units, mainly affecting females, beginning at 20 years of age [1]. Because of different clinical manifestations, the National Rosacea Society (NRS) mentions four rosacea subtypes, namely, erythematotelangiectatic, papulopustular, phymatous, and ocular [2]. Rosacea occurs mainly among Caucasians, although there are incidences within the Asian and African populations [1]. Since rosacea affects the face, it seriously affects patients' quality of life (QOL) and mental health [3, 4].

The pathogenesis of rosacea is still unclear [5]. However, it is certainly not attributable to a single pathogeny. Most previous studies suggest that seborrhea, *demodex* infestation, bacterial infection and mental factors may precipitate rosacea [5]. Mental factors are closely related to the pathogenesis and progression of rosacea [3]. A large-scale epidemiological survey found a close correlation between major depressive disorder and rosacea (OR: 4.81, 95% [1.39–16.62]) [6]. Moreover, the initial onset and/or subsequent flares, 60–90% of which are associated with emotional stress usually occur two days following stress [4]. Studies have confirmed the importance of psychological factors in the pathogenesis and progression of rosacea, and that it has a major psychosocial impact on patients [5].

Therefore, many suggest that, attention be paid to the assessment of rosacea patients' psychological and social function, in addition to physiological health [7]. The QOL questionnaire is a reference index for evaluating a patient's overall condition, prognosis, and treatment evaluation. Researchers should ideally use a disease-specific QOL questionnaire to accurately evaluate disease impact on QOL [8]. Commonly used disease-specific questionnaires in dermatology include the Dermatology Life Quality Index (DLQI) [9], the Skindex-29 [10], and the Skindex-16 [11]. These are used for many diseases, including psoriasis, chronic urticaria, and vitiligo. Research has shown that the instruments can assess both patients' social and psychological function and their disease status [9, 12]. However, each disease has its own characteristics; thus, some targeted QOL instruments have been developed for specific dermatoses. For example, the Chronic Urticaria Quality of Life Questionnaire (CU-Q2OL) is specifically for chronic urticaria [13], and the Cardiff Acne Disability Index (CADI) is specifically for acne [14]. In 2002, Kimbely et al. developed the rosacea-specific quality-of-life instrument, a targeted QOL instrument with sensitivity to the quality of life in rosacea patients [7].This instrument may help physicians and researchers measure QOL among rosacea patients [7]. However, it has not been tested on patients other than those in Kimberly N's research [7]. Testing across different racial groups may yield different results due to cultural variability in habits and other characteristics. Thus, the Rosacea-specific Quality-of-Life instrument (RosQol) must be tested across various countries and races.

Although rosacea is common among members of light-skinned racial groups [5], there are incidences of rosacea in China. Along with improving living standards, the Chinese pay more attention to facial dermatoses such as rosacea. This attention creates a need for a rosacea-specific QOL scale suitable for use in the Chinese population.

In our study, we translated the RosQol into Chinese, and evaluated its reliability and validity among 265 rosacea patients. Items 13 and 19 were not suitable for use in the Chinese population. These items were deleted, resulting in an adjusted Chinese version of the RosQol that is easy to complete and demonstrates acceptable validity, reliability, and sensitivity among the Chinese population.

## Materials and methods

### Ethics

The ethics review board of Xiangya Hospital Central South University approved the research. All study participants provided written informed consent. Participants agreed to the research contents. To protect the confidentiality and data of participants, all were assigned a unique identification number without identifying information.

### Subjects and study design

265 rosacea patients (220 women and 45 men) who received outpatient services at Xiangya Hospital of Central South University were recruited between March and November 2014. The inclusion criteria included the absence of any serious physical and mental illness. All patients met the necessary rosacea criteria [2]. Severity was determined by rosacea severity scores (RSSs), according to the NRS's standards [2]. Three dermatologists evaluated each patient by RSSs; we used an average of the three evaluations. The Dermatology Life Quality Index (DLQI) assesses QOL for most dermatoses, including rosacea [9, 12]. At their first clinical visit, patients provided their demographic and clinical information and completed the RosQol and the DLQI. Physicians provided therapy, skin care guidance, and patient education, among other services, based on patients' needs. 117 patients presented after a four-week treatment course for further data collection. The patients also specified the types of assessments that they completed. The data were also collected in the same date range.

### Questionnaire translation

We translated the RosQol from English into Chinese. Two native Chinese dermatologists, fluent in English, translated the Chinese version into English and merged the RosQol translations. A native, professional English translator reviewed the original and the back-translated English versions. During a panel meeting, the authors discussed ambiguous terms and decided on the final Chinese version. Five rosacea patients completed the near-final questionnaire and made suggestions, determining the final version.

The final Chinese-version RosQol comprised 21 items within three dimensions, including emotions (7 items), function (3 items), and symptoms (11 items). The response options were "never" (= 0), "seldom" (= 1), "sometimes" (= 2), "often" (= 3), and "always" (= 4) [7].

### Validity and reliability of the RosQol

We determined the validity and reliability of the Chinese-version RosQol. We assessed its internal consistency, using Cronbach's alpha, test-retest reliability, and Spearman's rank correlation. We also assessed its criterion-related and internal construct validity.

### Statistical analyses

The data were analyzed using SPSS (18.0), with p < 0.01 considered statistically significant.

## Results

### Subject characteristics

The mean age (± SD) of the 265 patients was 31.08 ± 10.728 (range: 15–80); the mean disease duration was 53.38 ± 57.560 months (range: 1–360). Table 1 shows detailed sample characteristics.

**Table 1:** Epidemiological data of rosacea patients.

Items	Results
Age (years), mean±SD (range)	31.08±10.728 (15–80)
Sex, n (%)	
Female	220 (83.0)
Male	45 (17.0)
Marital status, n (%)	
Single	113 (42.6)
Married	152 (57.4)
Education, n (%)	
Primary education and lower	4 (1.5)
Secondary education	106 (40.0)
Higher education	141 (53.2)
Advanced degrees	14 (5.3)
Age at onset (years), mean±SD (range)	26.18±11.388 (1–76)
Disease duration (months), mean±SD (range)	53.38±57.560 (1–360)
Disease duration, n (%)	
≤1 years	78 (29.4)
1–5 years	113 (42.6)
5–10 years	31 (11.7)
≥10 years	43 (16.2)

### Reliability analyses

The Chinese-version RosQol yielded a Cronbach's alpha of 0.935 (Table 2). Cronbach's alpha for the three dimensions ranged from 0.539 to 0.942 (Table 2). Cronbach's alpha for the individual items ranged from 0.929 to 0.937 (Table 3). Cronbach's alpha for items 13 and 19 was higher than that for the overall score, indicating that these items might not be sufficiently sensitive for use in the Chinese population. Therefore, we deleted these items and excluded them from the adjusted RosQol. Next, we determined the reliability and validity of the adjusted RosQol.

**Table 2:** Cronbach's alpha for the RosQol (n = 265).

	Total Score	Emotion	Symptoms	Function
Number of entries	21	11	7	3
Cronbach'sα coefficient	0.935	0.942	0.781	0.539

**Table 3:** Cronbach’s alpha for individual items of the RosQol (n = 265).

Item	Scale Mean if Item Deleted	Scale Variance if Item Deleted	Corrected Item-Total Correlation	Squared Multiple Correlation	Cronbach's Alpha if item deleted
1	40.03	266.711	0.678	0.630	0.931
2	40.94	278.552	0.456	0.279	0.935
3	39.93	265.594	0.656	0.520	0.931
4	39.90	266.750	0.705	0.629	0.931
5	40.20	270.719	0.560	0.395	0.933
6	39.90	270.649	0.576	0.443	0.933
7	40.13	260.021	0.803	0.765	0.929
8	40.19	258.105	0.822	0.801	0.928
9	39.78	263.204	0.713	0.581	0.930
10	40.31	259.919	0.765	0.676	0.929
11	39.82	261.149	0.746	0.621	0.930
12	40.69	260.691	0.746	0.677	0.930
13	41.35	276.972	0.389	0.277	0.936
14	39.81	263.880	0.755	0.611	0.930
15	39.83	275.503	0.408	0.353	0.935
16	39.95	273.447	0.444	0.271	0.935
17	39.89	273.103	0.546	0.474	0.933
18	40.06	270.449	0.582	0.503	0.933
19	40.98	279.776	0.341	0.231	0.937
20	39.95	262.286	0.788	0.677	0.929
21	40.23	271.711	0.471	0.395	0.935

Cronbach's alpha for the adjusted RosQol was 0.938; for the three dimensions, it ranged from 0.647 to 0.942 (Table 4). The Spearman-Brown correlation coefficient confirmed the instrument's reliability [15]. The Spearman-Brown correlation coefficient for the adjusted RosQol was 0.909; for the two parts, it ranged from 0.508 to 0.860.

**Table 4:** Cronbach's alpha for the adjusted RosQol (n = 265).

	Total Score	Emotion	Symptoms	Function
Number of entries	19	11	6	2
Cronbach's α coefficient	0.938	0.942	0.792	0.674
Spearman-Brown coefficient	0.909**	0.860**	0.508**	0.592**

**p<0.01

A total of 117 randomly selected patients were assessed again after the initial assessment (Table 4). The Pearson's correlation coefficient for each dimension ranged from 0.990 to 0.997; for the overall score, it was 0.997 (Table 5).

**Table 5:** Test-retest reliability for the adjusted RosQol ($\bar{x}$±S, n = 77).

	Total Score	Emotion	Symptoms	Function
T1	41.92±16.804	24.39±11.293	13.51±5.723	4.03±2.433
T2	41.62±16.271	24.22±10.954	13.42±5.596	3.99±2.354
R	0.997**	0.997**	0.996**	0.990**

**p<0.01

### Validity analyses

Construct validity: In our study, the item-total correlations ranged from 0.469 to 0.814 (Table 6). Moreover, the correlation coefficient between each of the three dimensions and the total score was 0.959, 0.857 and 0.577, respectively (Table 7). The dimensions of the scale proved to be in agreement, and each dimension was unique. Therefore, the adjusted Chinese-version RosQol showed good construct validity.

**Table 6:** Construct validity for the adjusted RosQol (n = 265).

	Emotion	Symptoms	Function	Total Score
Emotion	1			
Symptoms	0.723**	1		
Function	0.449**	0.445**	1	
Total Score	0.959**	0.857**	0.577**	1

**p<0.01

**Table 7:** Construct validity for individual items of the adjusted RosQol (n = 265).

Item	Total Score	Emotion	Symptoms	Function
1	0.715	0.777	0.530	0.304
2	0.501	0.434	0.562	0.250
3	0.697	0.738	0.518	0.291
4	0.738	0.788	0.552	0.308
5	0.608	0.632	0.468	0.281
6	0.621	0.556	0.703	0.262
7	0.828	0.864	0.639	0.357
8	0.846	0.879	0.637	0.416
9	0.749	0.693	0.774	0.371
10	0.797	0.831	0.587	0.370
11	0.779	0.808	0.593	0.357
12	0.780	0.810	0.552	0.401
14	0.784	0.789	0.638	0.413
15	0.469	0.359	0.360	0.861
16	0.505	0.398	0.630	0.302
17	0.591	0.465	0.752	0.327
18	0.626	0.485	0.776	0.349
20	0.814	0.834	0.621	0.432
21	0.531	0.420	0.411	0.876

Criterion-related validity: We used the DLQI as the gold standard, analyzing its correlation with the Chinese-version RosQol. A correlation coefficient of 0.686 was obtained (Table 8), indicating a good correlation between the two [16].

**Table 8:** Correlation with the DLQI (n = 251).

	Emotion	Symptoms	Function	Total Score
DLQI Score	0.706**	0.540**	0.361**	0.686**

**p<0.01

**Fig 1:**
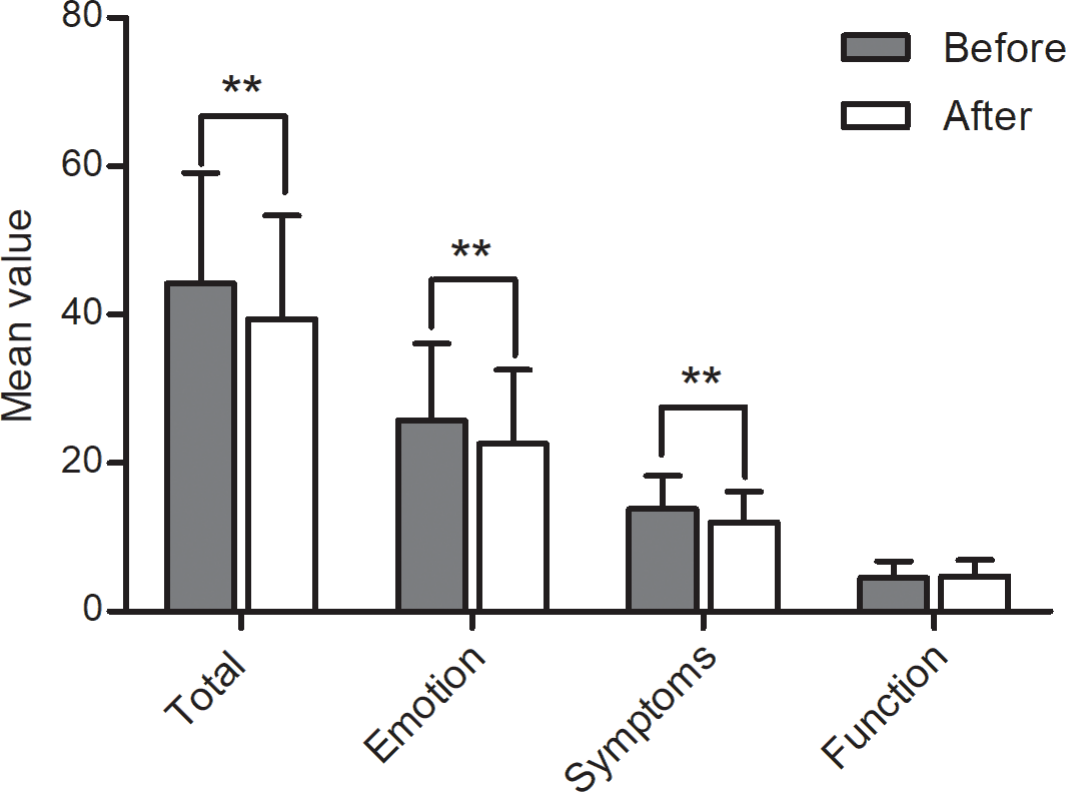
Rosacea: Pre- and post-treatment scores on the adjusted RosQol. After a four-week treatment course, there was a difference between pre- and post-treatment total and dimensional scores on the adjusted RosQol **p < 0.01.

**Fig 2:**
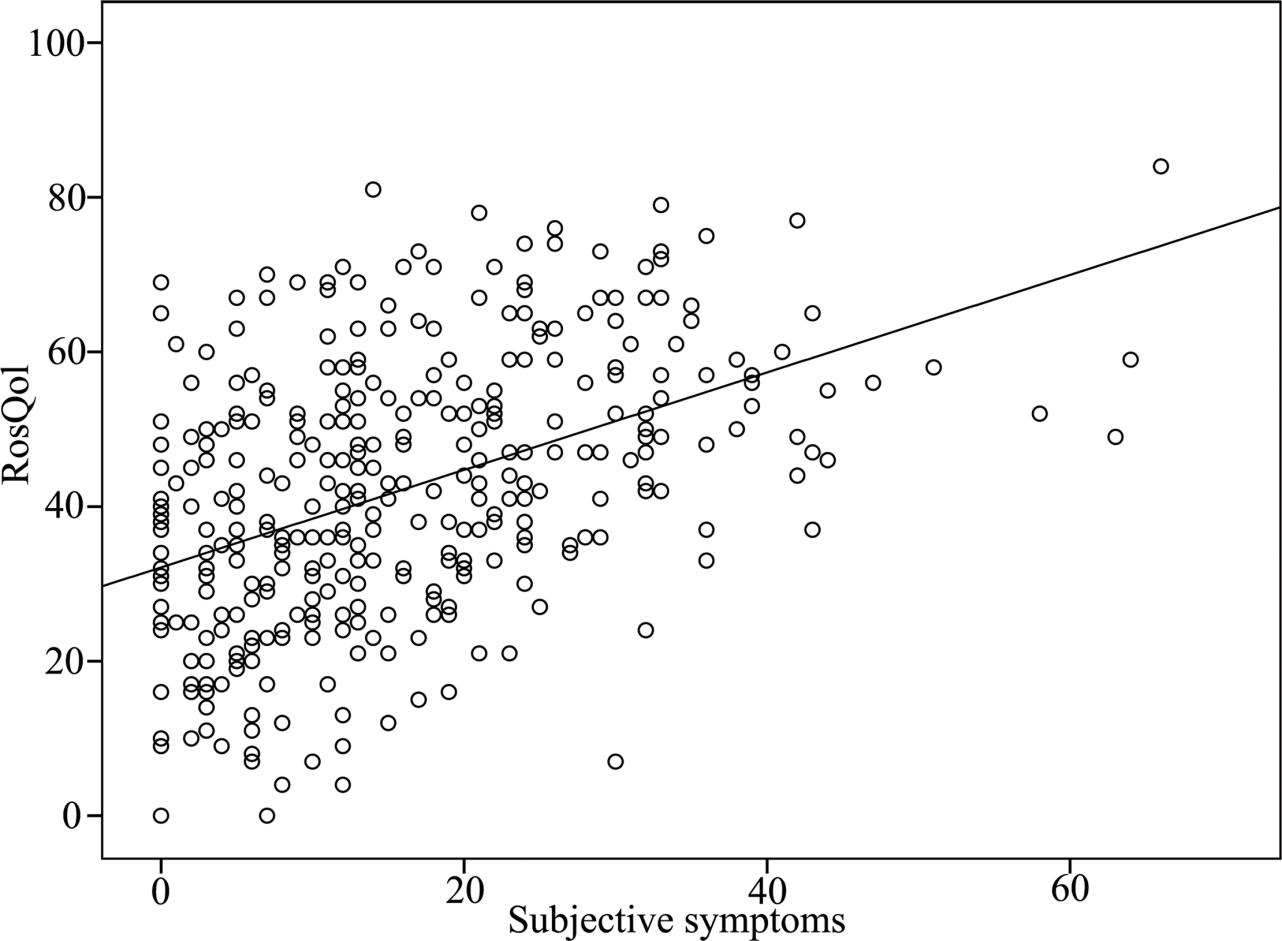
Rosacea: The relationship between self-evaluated symptoms and the adjusted RosQol. There was a positive correlation between the symptom score and adjusted RosQol score (r = 0.44, p < 0.01).

Discrimination validity and responsiveness: The measured QOL should change along with changes in patients' clinical conditions, thus reflecting the responsiveness of the relative instrument. [17]. In total, 117 (44.32%) patients completed the adjusted RosQol following a four-week treatment course. Post-treatment total and dimensional scores on the adjusted RosQol differed from the respective pre-treatment scores (Table 9, Fig 1). We evaluated patients' conditions using the total score for self-evaluated symptoms. The symptoms and RosQol scores correlated positively (r = 0.44, p < 0.01; Fig 2).

**Table 9:** Scores on the adjusted RosQol before and after treatment ($\bar{x}$±S, n = 117).

	Total Score	Emotion	Symptoms	Function
Before	44.20±14.933	25.79±10.295	13.85±4.431	4.56±2.199
After	39.44±13.897	22.64±9.938	12.05±4.133	4.74±2.210
T	5.944**	6.190**	5.197**	1.060

**p<0.01

## Discussion

Health-related quality of life (HRQoL) refers to the health status of individuals dealing with conditions such as injuries, medical interventions, and aging, in relation to environmental changes and subjective satisfaction with their economic situations, cultural backgrounds, and values [18]. Health status includes physical, psychological, and social aspects describing an individual's functional state. Along with subjective satisfaction, health status constitutes the predominant contributor to HRQoL. HRQoL must be measured through questionnaires. Such questionnaires are widely used for many skin diseases; the most commonly used include the DLQI, the Skindex-29, and the Skindex-16.

The RosQol was specifically developed for assessment of rosacea patients. However, the RosQol has been tested among patients in one study only; necessitating its validation on a different sample, as testing across different populations, might yield different results due to racial, cultural, and other differences.

In our research, we translated the RosQol into Chinese and verified its validity, reliability, and sensitivity among 265 Chinese rosacea patients.

First, we used Cronbach's alpha and Spearman's correlation to confirm the reliability and validity of the RosQol, respectively. In our results, the Cronbach's alpha for the total scale and psychological and symptoms dimensions exceeded 0.6, indicating good internal consistency [19]. Cronbach's alpha for the functional dimension indicated low internal consistency for that dimension.

We further calculated "Cronbach's alpha if item deleted." After items 13 and 19 were deleted, the Cronbach's alpha for the remaining items were below that of the total scale (0.935), indicating that the deleted items may not be suitable for the Chinese sample.

Item 13 is a member of the functional dimension. Skin care customs in the Chinese population led us to believe that, despite the growing use of makeup among Chinese women, the proportion of users in China is still significantly lower than that in the US, Europe, and other Asian countries including Japan and South Korea [20]. Due to awareness campaigns and other factors, many Chinese women consider cosmetics to be harmful to the skin, having the potential to cause facial symptoms. Due to the abovementioned reasons and Cronbach's alpha values, item 13 was considered unsuitable for use in the Chinese population.

Item 19 belongs to the symptoms dimension. Rosacea causes not only facial but also eye-related symptoms, which broadly include bloodshot eyes, abnormal sensations, burning, etc. Despite the 3–33% global incidence range of eye-related rosacea symtoms [21, 22, 23, 24], ocular symptoms were observed at a low (<7%) incidence rate in our study sample. These reasons prevented the accurate assessment of the symptoms scale, necessitating its assessment on a larger population.

Removal of items 13 and 19 resulted in the adjusted Chinese-version RosQol. Cronbach's alpha for the adjusted Chinese-version RosQol was 0.938; for the emotions, symptoms, and functional dimensions, it was 0.942, 0.792, and 0.674, respectively, indicating good internal consistency.

Based on the clinical characteristics of rosacea, the re-test interval was set three days after the initial test. A test-retest reliability value exceeding 0.07 is generally considered good. In our study, 77 (29.05%) patients were measured after the initial test; Pearson's correlation coefficient for the psychological, symptoms, and functional dimensions was 0.997, 0.996, and 0.990, respectively (p < 0.05). The test-retest reliability of the adjusted Chinese-version RosQol proved to be good.

In Spearman's rank correlation, a scale is divided into two parts, and the correlation coefficient for the two parts is obtained [15]. A correlation coefficient exceeding 0.7 generally indicates good internal consistency [15]. In our study, a value of 0.909 was obtained for the adjusted scale, suggesting good consistency between the items.

In summary, the adjusted RosQol showed good validity among Chinese patients. This result should ensure consistent results and relatively acceptable levels of random error under similar conditions.

We also examined construct and criterion-related validity of the adjusted RosQol. Construct validity comprises the evaluation of deviation between the measured value and the actual value of the target, and indicates the accuracy, validity, and correctness of a scale [25]. The item-total correlation exceeded 0.3, indicating good validity [25]. The correlation coefficients between the psychological, symptoms, functional dimensions and the total score were 0.959, 0.857 and 0.577, respectively. Moreover, the item-total correlations ranged from 0.469 to 0.846. The results show that the adjusted RosQol questionnaire has a reasonable structure, over the three dimensions, and good structural validity.

Criterion-related validity refers to the determination of the correlation between two scales, with one being generally considered a gold standard [16]. A correlation coefficient of 0.4–0.8 indicates good correlation [16]. Because no gold standard exists for assessing QOL in rosacea patients, we used the DLQI, which has good reliability and validity and has been tested on rosacea patients [4]. A Pearson's correlation coefficient of 0.686 between the two scales was obtained, and the Pearson's correction coefficients between the psychological, symptoms, functional dimensions and the DLQI were 0.706, 0.540, and 0.461, respectively. The criterion-related validity between the DLQI and the adjusted RosQol is thus good.

In summary, the construct validity and the criterion-related validity of the adjusted RosQol were good. Thus, this questionnaire reflects Chinese patients' QOL to a high degree.

We also determined the sensitivity of the adjusted RosQol. Sensitivity refers to the ability of the questionnaire to reflect changes in measured status before and after an intervention.

After their first visit, patients received comprehensive treatment, including drug treatment and physical therapy along with patient education. Of these patients, 117 (44.32%) were reassessed with the adjusted RosQol four weeks post-treatment. After treatment and symptoms improved, the total scores decreased. Similarly, scores on the psychological and symptoms dimensions decreased significantly after treatment, demonstrating the dimensions' sensitivity to change. Given the characteristics of rosacea, humid environments, high temperatures, and certain food could facilitate the onset or aggravate facial flushing [26]. During treatment, patients were given training in relevant life skills that increased their knowledge of rosacea [27]. Thus, they could effectively avoid exacerbating factors, leading to a higher functional dimension score. This result also demonstrated the scale's responsiveness.

We selected eight types of facial dermatitis symptoms on the basis of which patients could self-evaluate. Total scores on this measure reflected the severity of the patients' symptoms. We analyzed the correlation between the total symptom score and the adjusted RosQol score to determine whether the latter could reflect patients' condition. However, due to the complexity of rosacea presentation, most patients have more than one symptom that differs in severity [28]. This characteristic prohibits the use of summed scores for rosacea patients' individual symptoms to determine disease severity. Therefore, the RSSs are not suitable for assessing discriminant validity [28]. In our study, the scores for symptom evaluation and for the adjusted RosQol were positively related (r = 0.442, p < 0.01).

Overall, the adjusted RosQol has good reliability, validity, and responsiveness. It can effectively evaluate the impact of rosacea on the lives of Chinese patients, assess QOL among rosacea patients and clinical curative effects of existing treatments, and is a good indicator of health status. Chinese physicians and researchers could use this measure to monitor and understand the disease in its entirety and assess treatment efficacy among rosacea patients. The adjusted RosQol is also beneficial for treatment selection and evaluation. However, due to racial and cultural differences in habits and other characteristics, some items may not be suitable for assessment of all Chinese patients. Therefore, selection of suitable items for use in the Chinese population should continue to help develop a rosacea-specific QOL instrument for this population. In the past decades, advances in medical technology, especially in dermatology, have made the identification of rosacea possible. Furthermore, the public has more access to information about rosacea, contributing to the high incidence of rosacea. In summary, the adjusted RosQol is of vital importance to China.

## Supporting information

S1 File The original and chinese versions of rosacea-specific quality-of-life instrument. (DOCX)
