# Supplementary material for: The Rosacea-specific Quality-of-Life instrument (RosQol): Revision and validation among Chinese patients
Source: PLoS One. 2018 Feb 28;13(2):e0192487. doi: 10.1371/journal.pone.0192487 (PMC5831031; doi:10.1371/journal.pone.0192487)
Supplement: S1 File — (DOCX) [file pone.0192487.s001.docx]

S1 Table. The original version of rosacea-specific quality-of-life instrument

| RosaQoL items | Hypothesized construct |
| --- | --- |
| 1. I worry that my rosacea may be serious | Emotion |
| 2. My rosacea burns or stings | Symptom |
| 3. I worry about getting scars from my rosacea | Emotion |
| 4. I worry that my rosacea may get worse | Emotion |
| 5. I worry about side effects from rosacea medications | Emotion |
| 6. My rosacea is irritated | Symptom |
| 7. I am embarrassed by my rosacea | Emotion |
| 8. I am frustrated by my rosacea | Emotion |
| 9. My rosacea makes my skin sensitive | Symptom |
| 10. I am annoyed by my rosacea | Emotion |
| 11. I am bothered by the appearance of my skin (redness, blotchiness) | Emotion |
| 12. My rosacea makes me feel self-conscious | Emotion |
| 13. I try to cover up my rosacea (with makeup) | Functioning |
| 14. I am bothered by persistence/reoccurrence of my rosacea | Emotion |
| 15. I avoid certain foods or drinks because of my rosacea | Functioning |
| 16. My skin feels bumpy (uneven, not smooth, irregular) | Symptom |
| 17. My skin flushes | Symptom |
| 18. My skin gets irritated easily (cosmetics, aftershaves, cleansers) | Symptom |
| 19. My eyes bother me (feel dry or gritty) | Symptom |
| 20. I think about my rosacea | Emotion |
| 21. I avoid certain environments (heat, humidity, cold) because of my rosacea | Functioning |

附表2. 与玫瑰痤疮生活质量相关的特异性指标的中国改良版本

| 玫瑰痤疮生活质量相关指标 | 类别 |
| --- | --- |
| 1. 我担心玫瑰痤疮很严重 | 情感 |
| 2. 玫瑰痤疮发作我的脸会发热或刺痛 | 症状 |
| 3. 我担心玫瑰痤疮会留下疤痕 | 情感 |
| 4. 我担心我的玫瑰痤疮会加重 | 情感 |
| 5. 我担心玫瑰痤疮的药物治疗会有副作用 | 情感 |
| 6. 我的玫瑰痤疮易被刺激发作 | 症状 |
| 7. 我因为玫瑰痤疮感到尴尬 | 情感 |
| 8. 我因为玫瑰痤疮感到挫败 | 症状 |
| 9. 玫瑰痤疮使我的皮肤敏感 | 症状 |
| 10. 我常因玫瑰痤疮生气 | 情感 |
| 11. 我为我的皮肤感到困扰（发红、斑点） | 情感 |
| 12. 我总是感觉到我有玫瑰痤疮 | 情感 |
| 13. 玫瑰痤疮一直不消退或者反复发作很困扰我 | 情感 |
| 14. 因为玫瑰痤疮我不吃某些事物或饮品 | 功能 |
| 15. 我的皮肤很粗糙（不平、不光滑） | 症状 |
| 16. 我的皮肤时常潮红 | 症状 |
| 17. 我的皮肤易受到刺激（化妆品、爽肤水和洗面奶） | 症状 |
| 18. 我时常想到我的玫瑰痤疮 | 情感 |
| 19. 因为玫瑰痤疮我会避免接触一些环境因素（炎热、湿度大、寒冷） | 功能 |

S2 Table. The adjusted Chinese version of rosacea-specific quality-of-life instrument

| RosQoL items | Hypothesized construct |
| --- | --- |
| 1. I worry that my rosacea may be serious | Emotion |
| 2. My rosacea burns or stings | Symptom |
| 3. I worry about getting scars from my rosacea | Emotion |
| 4. I worry that my rosacea may get worse | Emotion |
| 5. I worry about side effects from rosacea medications | Emotion |
| 6. My rosacea is irritated | Symptom |
| 7. I am embarrassed by my rosacea | Emotion |
| 8. I am frustrated by my rosacea | Emotion |
| 9. My rosacea makes my skin sensitive | Symptom |
| 10. I am annoyed by my rosacea | Emotion |
| 11. I am bothered by the appearance of my skin (redness, blotchiness) | Emotion |
| 12. My rosacea makes me feel self-conscious | Emotion |
| 13. I am bothered by persistence/reoccurrence of my rosacea | Emotion |
| 14. I avoid certain foods or drinks because of my rosacea | Functioning |
| 15. My skin feels bumpy (uneven, not smooth, irregular) | Symptom |
| 16. My skin flushes | Symptom |
| 17. My skin gets irritated easily (cosmetics, aftershaves, cleansers) | Symptom |
| 18. I think about my rosacea | Emotion |
| 19. I avoid certain environments (heat, humidity, cold) because of my rosacea | Functioning |
